# Aging Process of Sea Salt Particles Driven by Glyoxal: Implications for Climate Effects

**DOI:** 10.3390/toxics14050415

**Published:** 2026-05-10

**Authors:** Yongpeng Ji, Zhiming Zhang, Shengping Chen, Qiuju Shi, Jiaxin Wang, Baocong Zhao, Weina Zhang, Jiangyao Chen, Yuemeng Ji

**Affiliations:** 1 Guangdong-Hong Kong-Macao Joint Laboratory for Contaminants Exposure and Health, Guangdong Key Laboratory of Environmental Catalysis and Health Risk Control, Institute Environmental Health and Pollution Control, Guangdong University of Technology, Guangzhou 510006, China; 2 Guangdong Basic Research Center of Excellence for Ecological Security and Green Development, Key Laboratory of City Cluster Environmental Safety and Green Development of the Ministry of Education, School of Environmental Science and Engineering, Guangdong University of Technology, Guangzhou 510006, China

**Keywords:** heterogeneous oxidation, reaction mechanisms, glyoxal, theoretical calculations, mixing processes

## Abstract

Atmospheric sea spray aerosol (SSA) undergoes chemical aging during long-distance transport, leading to significant alterations in its climate effects. However, the aging mechanisms of SSA driven by oxygenated volatile organic compounds (OVOCs) remain unclear. Hence, the aging processes of NaCl particles driven by glyoxal (GL), a representative OVOC, are systematically investigated using molecular dynamics (MD) simulations and density functional theory (DFT) calculations. MD simulations with high GL coverage show that GL readily mixes with NaCl and preferentially orients its carbonyl groups toward the NaCl surface. The adsorption of GL on the NaCl surface is dominated by the interaction between the O atom of GL (O_GL_) and the Na atom of the surface. DFT calculations with single GL coverage further reveal the formation of the O_GL_–Na bond between GL and NaCl. The mixing process of GL and NaCl is regulated by both the number of aldehyde groups engaging in the interfacial coordination and the corresponding lengths of O_GL_–Na bonds. The subsequent heterogeneous oxidation of GL by an OH radical proceeds mainly via a barrierless H-abstraction pathway to form HC(O)CO radicals, which may further react with methylamine/ammonia and contribute to brown carbon formation. Our results reveal the importance of incorporating such aging mechanisms into atmospheric models to improve climate predictions.

## 1. Introduction

Atmospheric sea spray aerosol (SSA) is one of the most abundant natural aerosol sources and plays a fundamental role in the global cycling of key elements, including sulfur, nitrogen, and halogens [1]. SSA, which is primarily generated through wind-driven wave breaking and bubble bursting at the ocean surface [2], is emitted into the atmosphere from oceans with an estimated global flux of 16,600 Tg y^−1^ [3]. The emitted SSA is dominated by inorganic salts, particularly sodium chloride (NaCl), and typically exhibits a core–shell structure with a crystalline NaCl core [4]. SSA significantly influences Earth’s radiation budget and global climate by directly scattering and absorbing solar radiation and indirectly acting as cloud condensation nuclei (CCN) or ice nuclei (IN) [2,5,6,7]. Once emitted, SSA particles can undergo long-distance transport, reaching inland regions [8]. During this process, SSA serves as an important reactive interface for heterogeneous chemical processes of atmospheric species [4]. Moreover, the mixing of atmospheric species with SSA, along with subsequent interfacial reactions on SSA, drives the chemical aging of SSA. This process modifies the chemical composition, morphology, and mixing state of SSA, resulting in pronounced changes in CCN activity and optical properties [4,9,10,11,12]. Hence, the environmental and climatic effects of SSA are significantly dependent on its atmospheric aging.

Previous studies [4,10,11,13,14,15,16,17,18] have demonstrated that SSA can mix with a wide range of atmospheric components during long-distance transport, including inorganic and organic compounds, dusts, heavy metals, and volcanic aerosols, resulting in alterations of its physiochemical properties. For example, a field observation shows that the mixing of sea salt particles with mineral dust during dust periods results in a reduction in their hygroscopicity [10]. In addition, deliquescence-mode experiments with mixed aerosol particles of NaCl and MgSO_4_ reveal that the Mg salts (MgSO_4_ and the formed MgCl_2_) that are enriched at the particle surface can lower the deliquescence point of NaCl particles [17]. Notably, organic species play a significant role in the aging of SSA. A modeling study has reported that hygroscopic growth of SSA is suppressed by 4–20% via the internal mixing with organic compounds under high relative humidity, and light scattering is also reduced by up to 37%, leading to an attenuation of the radiative cooling effect of SSA [11]. In addition, an experimental study by Ghorai et al. has revealed that when mixed with malonic acid and glutaric acid, cubic crystalline NaCl is modified into rounded particles, and its hygroscopic growth is suppressed [15]. Hence, the mixing of organic compounds with SSA, along with their subsequent heterogeneous reactions, plays a critical role in determining the physicochemical properties of SSA.

Oxygenated volatile organic compounds (OVOCs), which represent an important class of organic species due to their high abundance and reactivity, are primarily emitted from vehicular exhaust and secondary atmospheric chemical processes [19]. In addition, they can be introduced into the atmosphere through emissions from biological sources [20], hydrocarbon oxidation [21], the evaporation of oxygenated solvents or fuels, as well as incomplete combustion from mobile and stationary hydrocarbon-fueled sources [22]. Previous studies have shown that the photooxidation of OVOCs plays an important role in the formation of secondary organic aerosols (SOA) and tropospheric ozone (O_3_) [23,24,25,26,27]. Glyoxal (GL) is one of the most abundant and reactive OVOCs in the atmosphere. Owing to its high reactivity and water solubility, GL can easily enter the particle phase and plays a crucial role in the formation of SOA [28,29,30,31]. Except for the reaction in the gas phase [32,33] and aqueous phase [34,35,36,37,38], the heterogeneous reaction of GL on atmospheric particles [39,40,41,42,43,44] is an important sink of GL. Some studies have shown that the mixing of GL with mineral particles has formed oligomers, organosulfates and organic acid on mineral particles, which altered the optical properties, particle size and hygroscopicity of mineral particles [39,45]. However, the aging mechanism of SSA driven by GL remains unclear.

Hence, we performed a theoretical approach to investigate the GL-driven aging processes of a NaCl particle, the dominant component of SSA. Using molecular dynamics (MD) simulations, the mixing behavior of NaCl particles with GL was elucidated. GL-driven adsorption modes on the NaCl particles were characterized by density functional theory (DFT) calculations. Charge density difference (CDD) analysis was performed to reveal the nature of the interfacial interaction between GL and NaCl and the mixing mechanisms. In addition, the heterogeneous oxidation mechanisms of GL by OH radicals were further established and the implications of the GL-driven aging processes of NaCl on the atmosphere were discussed.

## 2. Computational Methods

### 2.1. Molecular Dynamics Simulations

All classical MD simulations were performed using the large-scale atomic/molecular massively parallel simulator (LAMMPS) package [46]. Since NaCl constitutes the dominant component (approximately 90%) of fresh SSA [1] and is widely used as a representative of SSA [47,48], NaCl (001) was selected as a simplified model of SSA in this work. A (18 × 18) NaCl (001) slab model (denoted as NaCl hereinafter) with 10 atomic layers was constructed for MD simulations. The NaCl (001) slab was centered in the simulation box with dimensions of 97 × 97 × 122 Å^3^ in x, y, and z directions, respectively. A total of 25 gaseous GL molecules were inserted into the vacuum region of the simulation box to construct the mixing system of NaCl (001) and GL according to the ideal gas equation (Equation (1)):

(1) *pV* = *nRT*

where *p*, *V*, *n*, *R* and *T* represent pressure, volume, number of gaseous molecules, the universal gas constant and temperature, respectively. A 5.0 ns MD simulation with the time step of 1.0 fs was carried out in the isothermal–isobaric (*NPT*) ensemble (*P* = 1 atm, *T* = 298.15 K) using the Nosé–Hoover thermostat and barostat, respectively, to guarantee the thermodynamic equilibrium of each mixing system [49,50]. NaCl was described from Joung and Cheatham parameters [51], while GL was described by the General Amber Force Field (GAFF) force field [52] with the restrained electrostatic potential (RESP) charge. The periodic boundary conditions were applied in all three dimensions. A cut-off distance of 12.0 Å was used for the short-range interactions. The long-range electrostatic interactions were computed using the particle–particle–particle–mesh (PPPM) summation algorithm with a dimensionless accuracy of 1 × 10^−5^ [53]. The analyses of the atomic density profiles were calculated according to Equation (2):

(2) $$\rho (z)=\frac{N(∆z)}{V}$$

where *ρ*(*z*) is the density in the *z* direction, *N*(Δ*z*) is the average number of atoms in a small bin of thickness Δ*z* in the *z*-axis of the simulation box and *V* denotes the volume of the simulation box.

### 2.2. Density Function Theory Calculations

All DFT calculations were performed using the Vienna ab initio simulation package (VASP, version 5.4.4) [54]. A (3 × 3) NaCl (001) surface with a four-layer slab was constructed to simulate the sea salt surface. For all calculations, the Perdew–Burke–Ernzerhof (PBE) [55] generalized gradient approximation (GGA) exchange–correlation functional was used to treat the electron interactions. The core electrons were described with the projector augmented wave (PAW) method [56]. The plane-wave kinetic energy cutoff was set to 400 eV, and the convergence criterion of structural optimization was −0.01 eV/Å. The vacuum region was set to approximately 30 Å to eliminate the interlayer interactions. A 2 × 2 × 1 Monkhorst–Pack k-point grid was applied. The dispersion corrections were considered using the DFT-D3(BJ) scheme [57,58]. The climbing nudged elastic band method [59] and the improve dimer methods [60] were performed to confirm the transition states (TSs) with the convergence criterion of −0.05 eV/Å. In this work, the adsorption energy (Δ*E*) is defined as Δ*E* = *E*_(NaCl-GL)_ − (*E*_NaCl_ + *E*_GL_), where *E*_(NaCl-GL)_ represents the total energy of the mixing system and *E*_NaCl_ and *E*_GL_ are the energies of NaCl and GL, respectively. The energy barrier is defined as *E*_TS_ − *E*_IS_, and reaction energy is defined as *E*_FS_ − *E*_IS_, where *E*_TS_, *E*_IS_ and *E*_FS_ represent the energies of the TSs, initial states (ISs), and final states (FSs) in the corresponding pathways, respectively. CDD was calculated to investigate the charge transfer between GL and the NaCl surface, which is defined as the difference between the charge densities of the mixing system of GL-NaCl and the sum of the charge densities of the isolated GL and the NaCl surface. CDD results were visualized using the VESTA package [61]. Considering the compositional complexity of real SSA, other inorganic ions were not explicitly included in the present model, which may introduce certain limitations. However, this simplified model provides a fundamental basis for understanding the mixing of GL and SSA at the molecular level.

## 3. Results and Discussion

### 3.1. Mixing Processes of GL with NaCl

**Figure 1:**
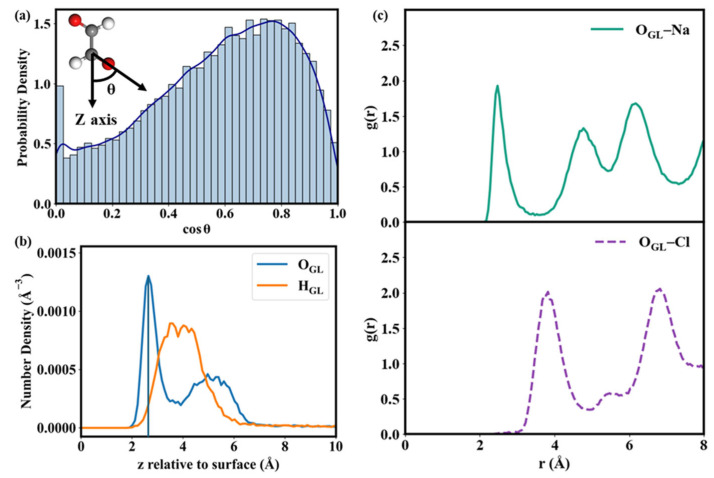
(**a**) Molecular orientation distributions of GL molecules on NaCl surfaces. (**b**) Atomic density profiles (ADPs) of GL as a function of distance from NaCl surfaces. *z* = 0 Å are the mean positions for the Na atoms of the NaCl surface. The locations of the ADP peaks represent the average distance between the corresponding atoms and NaCl surfaces. (**c**) Radial distribution functions (RDFs) for the possible atomic pairs between O_GL_ and NaCl surface.

The mixing process of GL with NaCl, which represents the initial step of the GL-driven aging of NaCl, was simulated using MD simulations. Several representative snapshots of the mixing process of GL with NaCl shown in Figure S1 reveal that GL readily adsorbs onto the NaCl surface and exhibits distinct orientations, leading to the formation of different adsorption configurations. To systematically characterize these adsorption configurations, the orientation of GL relative to the surface was quantified by using the cos(θ) value, where θ is defined as the angle between the *z*-axis (perpendicular to the surface) and the carbonyl group of GL. A value of 0 corresponds to the carbonyl group being completely parallel to the NaCl surface, while a value of 1 indicates a perpendicular orientation with the carbonyl group pointing toward the surface. The probability distributions of cos(θ) are shown in Figure 1a. A broad maximum centered around 0.8 and a noticeable population at 0 are observed, corresponding to configurations in which the carbonyl group points to the surface and lies parallel to the surface, respectively. The relatively broad distribution around 0.8 suggests that the carbonyl group is predominantly oriented toward the NaCl surface, implying that it may serve as the active site of GL to interact with NaCl.

To clarify the main active site of GL interacting with the NaCl surface, atomic density profiles (ADPs) of GL were calculated as functions of the distance normal to the NaCl surface and are shown in Figure 1b. Analysis of ADPs indicates that the interaction between GL and the NaCl surface is predominantly driven by the O atoms of GL (O_GL_), as evidenced by the positions of the ADP peak centers for O_GL_ and H_GL_ at ~2.5 Å and ~4.0 Å, respectively. The shorter distance of the O_GL_ to the surface suggests that O_GL_ serves as the primary interaction site of GL to interact with NaCl. Hence, the possible atomic pairs involved in the interaction between O_GL_ and the NaCl surface, i.e., O_GL_–Na and O_GL_–Cl, are further examined by radial distribution functions (RDFs), which quantify the probability of finding a given atom at a distance *r* from a reference atom. As delineated in Figure 1c, the RDF for O_GL_–Na exhibits a pronounced first peak at approximately 2.5 Å, while the first peak of O_GL_–Cl occurs at a longer distance of approximately 4.2 Å, with a comparable RDF intensity to that of O_GL_–Na. It indicates a stronger interaction between O_GL_ and Na atoms relative to that between O_GL_ and Cl atoms. Hence, GL can be effectively adsorbed on the NaCl surface, which is predominantly driven by the interaction between O_GL_ and Na atoms.

### 3.2. Mixing Mechanisms of GL with NaCl

**Figure 2:**
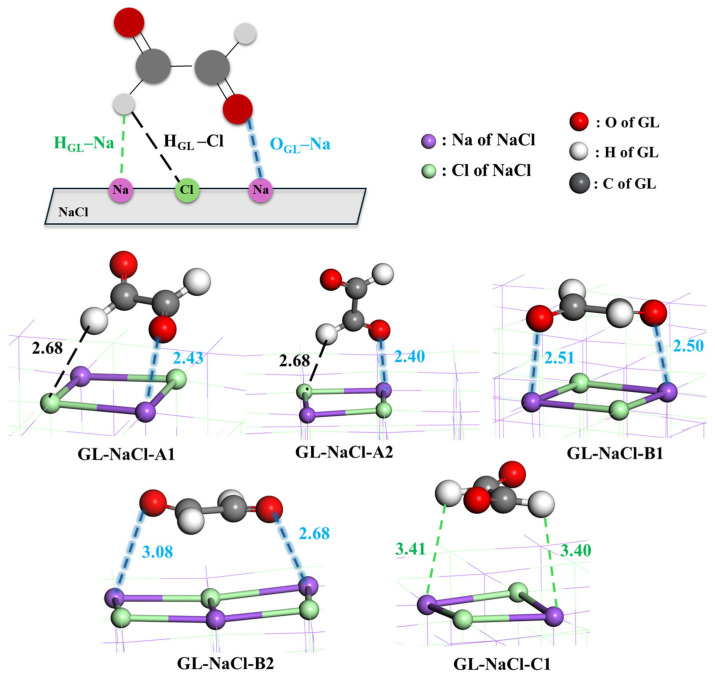
Optimized geometries for adsorption configurations of GL on the NaCl surface along with the scheme of bonds formed between GL and NaCl in adsorption configurations (bond length in Å).

**Figure 3:**
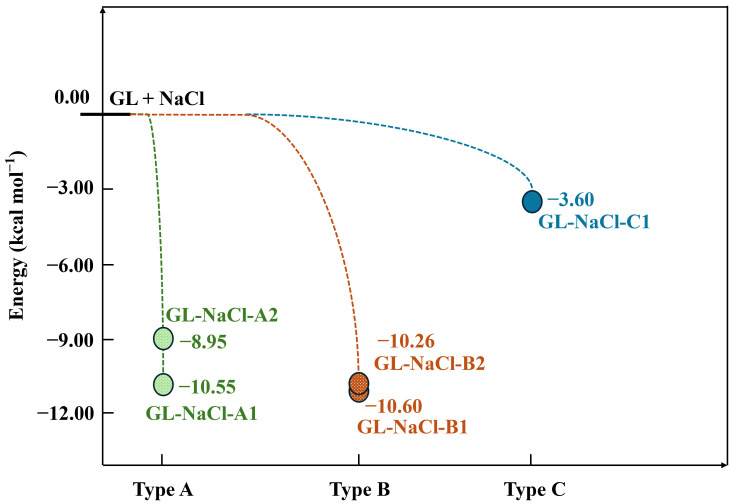
Potential energy surfaces for mixing pathways of GL with NaCl.

To understand the mixing mechanisms of GL with NaCl at the atomic level, adsorption configurations of GL on the NaCl surface were identified using DFT calculations. The optimized geometries of all adsorption configurations are presented in Figure 2 and Figure S2, while the potential energy surfaces of all possible mixing pathways are shown in Figure 3 and Figure S3. Depending on the number of O_GL_–Na bonds formed in adsorption configurations, three types of interaction modes were identified: Type A, where GL is adsorbed on NaCl with the formation of one O_GL_–Na bond; Type B, where GL is adsorbed on NaCl with the formation of two O_GL_–Na bonds; and Type C, where GL is adsorbed on NaCl without forming an O_GL_–Na bond. For Type A, five adsorption configurations were identified. Except for the O_GL_–Na bond, there exists an extra H_GL_–Cl bond between GL and NaCl in GL-NaCl-A1/2, with the bond length of 2.68 Å (Figure 2). The lengths of the O_GL_–Na bonds in GL-NaCl-A1 and GL-NaCl-A2 are 2.43 and 2.40 Å, respectively, suggesting a slightly stronger interaction between the O_GL_ and Na atoms in GL-NaCl-A2. However, the Δ*E* of GL-NaCl-A2 with −8.95 kcal mol^−1^ is 1.60 kcal mol^−1^ higher than that of GL-NaCl-A1 (Figure 3), indicating a stronger interaction between GL and NaCl in GL-NaCl-A1 than that in GL-NaCl-A2. This is attributed to the number of aldehyde groups of GL involved in the interaction. As shown in Figure 2, GL interacts with NaCl via both aldehyde groups in GL-NaCl-A1, while a single aldehyde group of GL participates in the interaction in GL-NaCl-A2. Similar results can also be drawn in three other adsorption configurations (Figure S2). The Δ*E* of GL-NaCl-A5, where GL interacts with NaCl via a single aldehyde group, is calculated to be −8.86 kcal mol^−1^, which is at least 1.64 kcal mol^−1^ higher than those of GL-NaCl-A3 and GL-NaCl-A4 (Figure S3).

For Type B, two adsorption configurations were identified. In GL-NaCl-B1, two O_GL_–Na bonds with bond lengths of 2.50 and 2.51 Å are formed, which are both shorter than those in GL-NaCl-B2 (Figure 2), indicating a stronger interaction between GL and NaCl in GL-NaCl-B1. The Δ*E* of GL-NaCl-B1 (−10.60 kcal mol^−1^) is 0.34 kcal mol^−1^ lower than that of GL-NaCl-B2 (Figure 3), further supporting the correlation between shorter O_GL_–Na bond lengths and stronger interaction strength. Compared the dominant configuration between Type A and Type B, the Δ*E* of GL-NaCl-B1 is only 0.05 kcal mol^−1^ lower than that of GL-NaCl-A1 (Figure 3). The difference in Δ*E* is smaller than the thermal energy at ambient temperature, indicating that the contributions of Type A and Type B configurations to the mixing of GL and NaCl should not be evaluated solely based on adsorption energies. As revealed by the broad cos(θ) distribution in the MD simulations (Figure 1a), GL molecules exhibit a wide range of interfacial orientations. Type B configurations require the simultaneous formation of two O_GL_–Na bonds and thus impose stronger orientational constraints, while Type A configurations retain greater rotational and configurational freedom. This indicates that although GL-NaCl-A1 has a higher adsorption energy than GL-NaCl-B1, Type A configurations remain competitive.

For Type C, only one adsorption configuration is identified. As shown in GL-NaCl-C1 (Figure 2), no O_GL_–Na bond is formed between GL and NaCl, but two O_GL_–Cl bonds are formed, with the bond lengths of 3.40 and 3.41 Å. The corresponding Δ*E* of GL-NaCl-C1 is at least 5.35 kcal mol^−1^ higher than those of the adsorption configurations in Type A and B, indicating that this interaction mode is of minor importance for the mixing of GL and NaCl (Figure 3). This result is consistent with MD simulations, which show that the interaction between O_GL_ and Na dominates the mixing of GL and NaCl.

**Figure 4:**
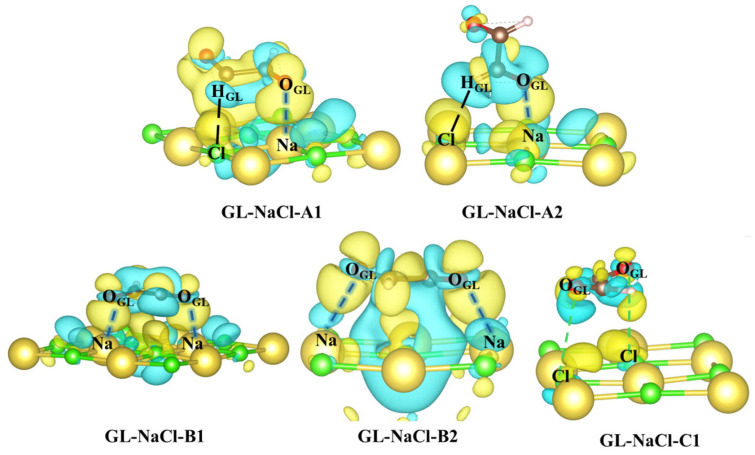
Charge density difference for adsorption configurations of GL on the NaCl surface, where the yellow and blue parts represent the area gaining and losing electrons, respectively.

To further elucidate the nature of the interaction between GL and NaCl, CDD analysis of the above configurations was performed, and the results are shown in Figure 4. Except for GL-NaCl-C1, all configurations clearly exhibit the pronounced electron accumulation around the O_GL_ and the corresponding electron depletion around the surface Na atom, indicating significant charge transfer from the Na atom to the O_GL_ atom. However, GL-NaCl-C1, without the formation of an O_GL_–Na bond, shows a weak charge transfer. It is consistent with the weaker interaction between GL and NaCl, confirming that such interactions play a minor role in the mixing process. For Type A, GL-NaCl-A1, in which both aldehyde groups participate in the interactions, exhibits more extensive charge transfer than GL-NaCl-A2, where only one aldehyde group is involved. It suggests that the number of aldehyde groups participating in the interaction plays a dominant role in regulating the mixing process. Similarly, for Type B, the charge transfer between O_GL_ and the Na atom in GL-NaCl-B1 is stronger than that in GL-NaCl-B2, in line with the shorter O_GL_–Na bonds. Hence, the mixing of GL and NaCl is regulated by the number of aldehyde groups that participate in the interaction and the bond lengths of O_GL_–Na.

### 3.3. Heterogeneous Oxidation Mechanisms of GL on NaCl Surface

**Figure 5:**
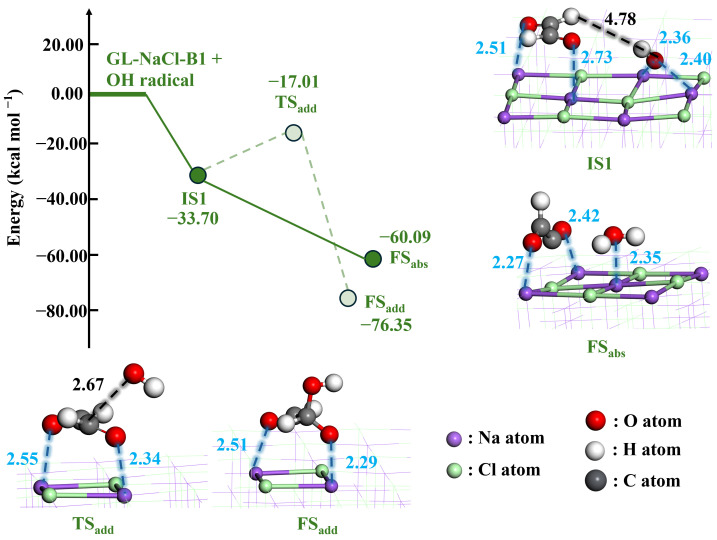
The potential energy surfaces of reaction pathways of GL and the OH radical along with the optimized geometries of key stationary points (bond lengths in Å).

In the following section, we investigate the reaction of GL with an OH radical, which represents a key step in the aging process of NaCl. The potential energy surfaces of all possible pathways are presented in Figure 5, along with the optimized geometries of key stationary points. Starting from the most stable adsorption configuration of GL on NaCl (GL-NaCl-B1), the mixing of an OH radical with GL-NaCl-B1 was further explored to identify the initial states (IS) in the oxidation of GL. Several ISs were identified, and their optimized geometries are listed in Figure S4 along with the corresponding Δ*E*. As shown in IS1 of Figure 5, the O atom of the OH radical (O_OH_) interacts with the Na atom of the NaCl surface, forming two O_OH_–Na bonds with bond lengths of 2.36 and 2.40 Å. The energy for the formation of IS1 is −33.70 kcal mol^−1^, indicating a strong interaction between the OH radical and the NaCl surface.

Subsequently, the reaction of GL with the OH radical proceeds via two pathways, that is, the H-abstraction (R_abs_) and OH-addition (R_add_) pathways. For the H-abstraction pathway, the OH radical approaches the H atom of GL (H_GL_), and H_GL_ is subsequently abstracted by the OH radical accompanied by the breaking of the C–H_GL_ bond. This process is barrierless. For the OH-addition pathway, the addition of the OH radical to the C atom of GL (C_GL_) forms a C_GL_–O_OH_ bond. However, the OH-addition pathway proceeds via a TS, with an energy barrier of 16.69 kcal mol^−1^. This is because the OH radical must overcome its interaction with the NaCl surface and break two O_OH_–Na bonds in IS1. Hence, R_abs_ is the dominant pathway for the reaction between GL and the OH radical, and the HC(O)CO radical is predicted to be the dominant product. To evaluate the possible influence of entropic effects, Gibbs free energy corrections were performed for the key stationary points at 298.15 K. Gibbs free energy profiles for the reaction pathway of GL and the OH radical are shown in Figure S5. The free energy barrier of R_add_ is 15.19 kcal mol^−1^, whereas R_abs_ remains barrierless. Therefore, R_abs_ is the dominant pathway after considering entropic effects.

According to the previous study [40], the HC(O)CO radical is more reactive toward the reaction with methylamine and ammonia than GL. This radical reacts with methylamine/ammonia to form aminoalcohol radical intermediates, which are rapidly oxidized by O_2_ to produce imine and diimine derivatives. These derivatives are important precursors of brown carbon. The barrierless formation of the HC(O)CO radical from the reaction of GL on NaCl implies that GL-driven aging processes are expected to significantly alter the optical properties of NaCl. In addition, the previous experimental studies also support the atmospheric uptake and reaction of GL on NaCl. Corrigan et al. [62] observed the RH-dependent uptake of GL by NaCl aerosol, suggesting that NaCl aerosol can serve as a reactive interface for GL. Shapiro et al. [63] observed high-molecular-weight secondary organic products in the mixing of GL and aqueous NaCl. Therefore, GL-driven aging represents an important atmospheric evolution process of sea salt particles. In the present study, the DFT calculations were performed using a single GL molecule on the NaCl surface to identify the interaction mechanism between GL and NaCl. Therefore, the effects of high GL coverage, as represented in MD simulations, were not considered in the DFT calculations. Under high coverage conditions, the adsorption mode of GL on NaCl can be influenced by intermolecular interactions among GL molecules, steric effects, and the competition for surface active sites. Hence, the influences of high GL coverage on the mixing between GL and NaCl remain a limitation of this study and should be further considered in future work. However, the dominant O_GL_–Na interaction identified by DFT is consistent with the ADP and RDF results from MD simulations, indicating that the DFT calculations have captured the key interaction between GL and NaCl.

## 4. Conclusions and Atmospheric Implications

Atmospheric sea spray aerosol (SSA), one of the most abundant natural aerosol types, undergoes chemical aging during long-distance transport, leading to modifications of its chemical composition and physicochemical properties. However, the molecular-level aging mechanisms of SSA driven by OVOCs remain poorly understood. In this work, we investigated the aging processes of NaCl particles driven by GL, a representative OVOC, by using MD simulations and DFT calculations. MD simulation results reveal that GL can effectively mix with NaCl, with its carbonyl groups pointing toward the surface. DFT calculations identify the dominant interaction between O atoms of GL and Na atoms of NaCl surface, mediated by the formation of O_GL_–Na bonds. Charge density difference analysis shows the electron transfer from surface Na atoms to the O atoms of GL, confirming the formation of strong interfacial interactions. The strength of the interaction between GL and NaCl is regulated by two key factors: the number of aldehyde groups involved in the interaction and the lengths of O_GL_–Na bonds. Subsequently, the heterogeneous oxidation of GL by the OH radical proceeds via two competing pathways: the H-abstraction and OH addition pathways. The H-abstraction pathway is barrierless, whereas the OH-addition pathway requires an energy barrier of 16.69 kcal mol^−1^. Thus, the H-abstraction pathway is the dominant pathway for the reaction of GL and the OH radical, forming the HC(O)CO radical. The HC(O)CO radical is highly reactive and can further undergo hydration and reaction with methylamine and ammonia to form light-absorbing compounds, thereby altering the radiative effects of SSA. Our results highlight the importance of considering GL-driven aging processes when investigating the physicochemical properties and atmospheric implications of sea salt particles. The present simulations were performed under dry conditions. In the atmosphere, variable relative humidity may influence SSA surface water coverage, ion mobility, and interfacial reactivity. Surface water molecules may compete with GL for surface active sites, weaken O_GL_–Na interactions, or promote GL hydration. In addition, water molecules may affect the stabilization of OH radicals and transition states, thereby altering the contribution of the H-abstraction and OH-addition pathways to the reaction of GL and the OH radical. Therefore, future studies using hydrated NaCl surfaces or deliquesced sea salt models are needed to further evaluate the influence of relative humidity on GL-driven aging.

## Data Availability

The raw data supporting the conclusions of this article will be made available by the authors on request.

## Supplementary Materials

The following supporting information can be downloaded at https://www.mdpi.com/article/10.3390/toxics14050415/s1: Figure S1. Selected snapshots for the MD trajectories of uptake process of GL onto NaCl surface; Figure S2. Optimized geometries for adsorption configurations of GL on NaCl (bond lengths in Å); Figure S3. Potential energy surfaces for mixing pathways of GL with NaCl; Figure S4. Potential energy surfaces for the formation of ISs along with the corresponding optimized geometries (bond lengths in Å); Figure S5. Gibbs free energy profiles for reaction pathways of GL and OH radical.
