# Supplementary material for: Aging Process of Sea Salt Particles Driven by Glyoxal: Implications for Climate Effects
Source: Toxics. 2026 May 10;14(5):415. doi: 10.3390/toxics14050415 (PMC13211330; doi:10.3390/toxics14050415)
Supplement: Supplementary file 1 [file toxics-14-00415-s001.zip › toxics-4264784-supplementary.pdf]

Supporting information:

Aging process of sea salt particles driven by glyoxal: implications for climate effects

Yongpeng Ji[a,b], Zhiming Zhang[a,b], Shengping Chen[a,b], Qiuju Shi[a,b], Jiaxin Wang[a,b], Baocong Zhao[a,b], Weina Zhang[a,b], Jiangyao Chen[a,b], Yuemeng Ji[a,b,*]

a Guangdong-Hong Kong-Macao Joint Laboratory for Contaminants Exposure and Health, Guangdong Key Laboratory of Environmental Catalysis and Health Risk Control, Institute Environmental Health and Pollution Control, Guangdong University of Technology, Guangzhou 510006, China.

b Guangdong Basic Research Center of Excellence for Ecological Security and Green Development, Key Laboratory of City Cluster Environmental Safety and Green Development of the Ministry of Education, School of Environmental Science and Engineering, Guangdong University of Technology, Guangzhou, 510006, China.

*Corresponding author: **Prof. Yuemeng Ji**

E-mail address: jiym@gdut.edu.cn

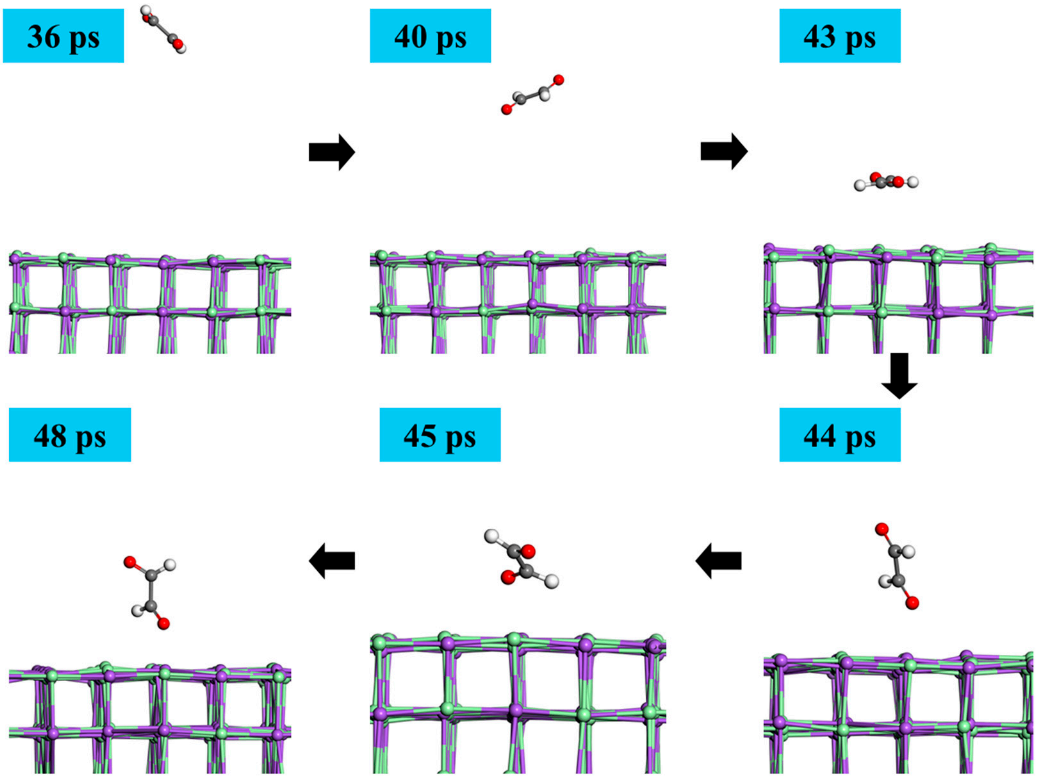

Figure S1. Selected snapshots for the MD trajectories of uptake process of GL onto NaCl surface.

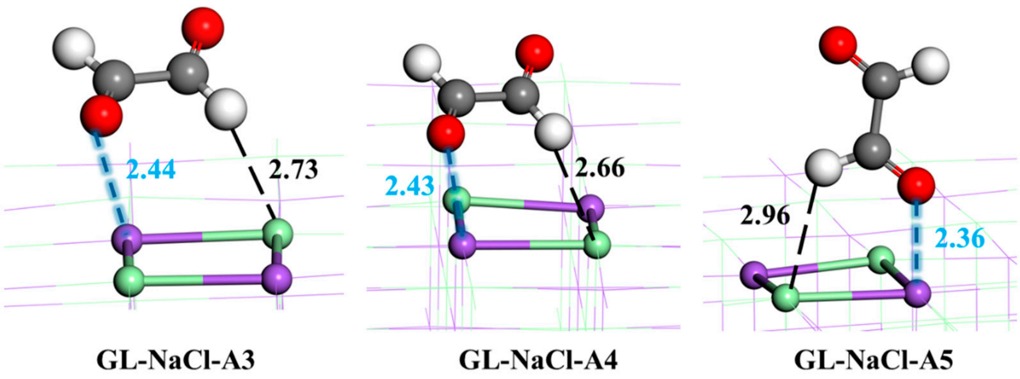

Figure S2. Optimized geometries for adsorption configurations of GL on NaCl (bond lengths in Å).

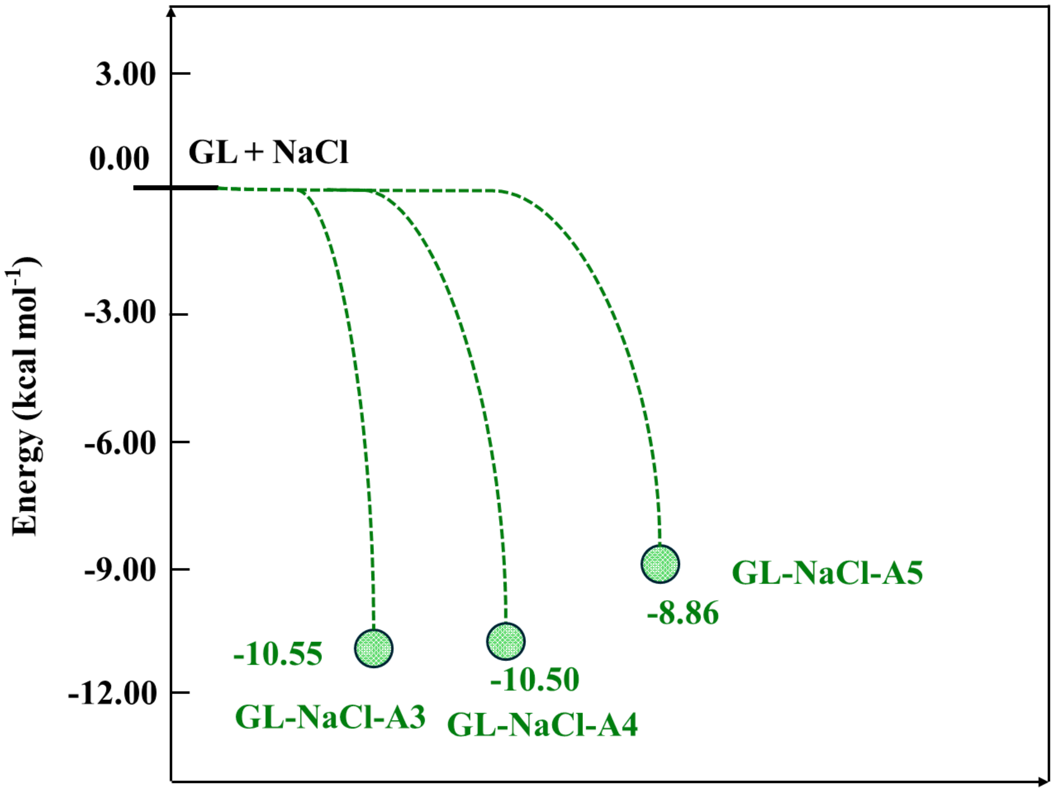

Figure S3. Potential energy surfaces for mixing pathways of GL on NaCl surface.

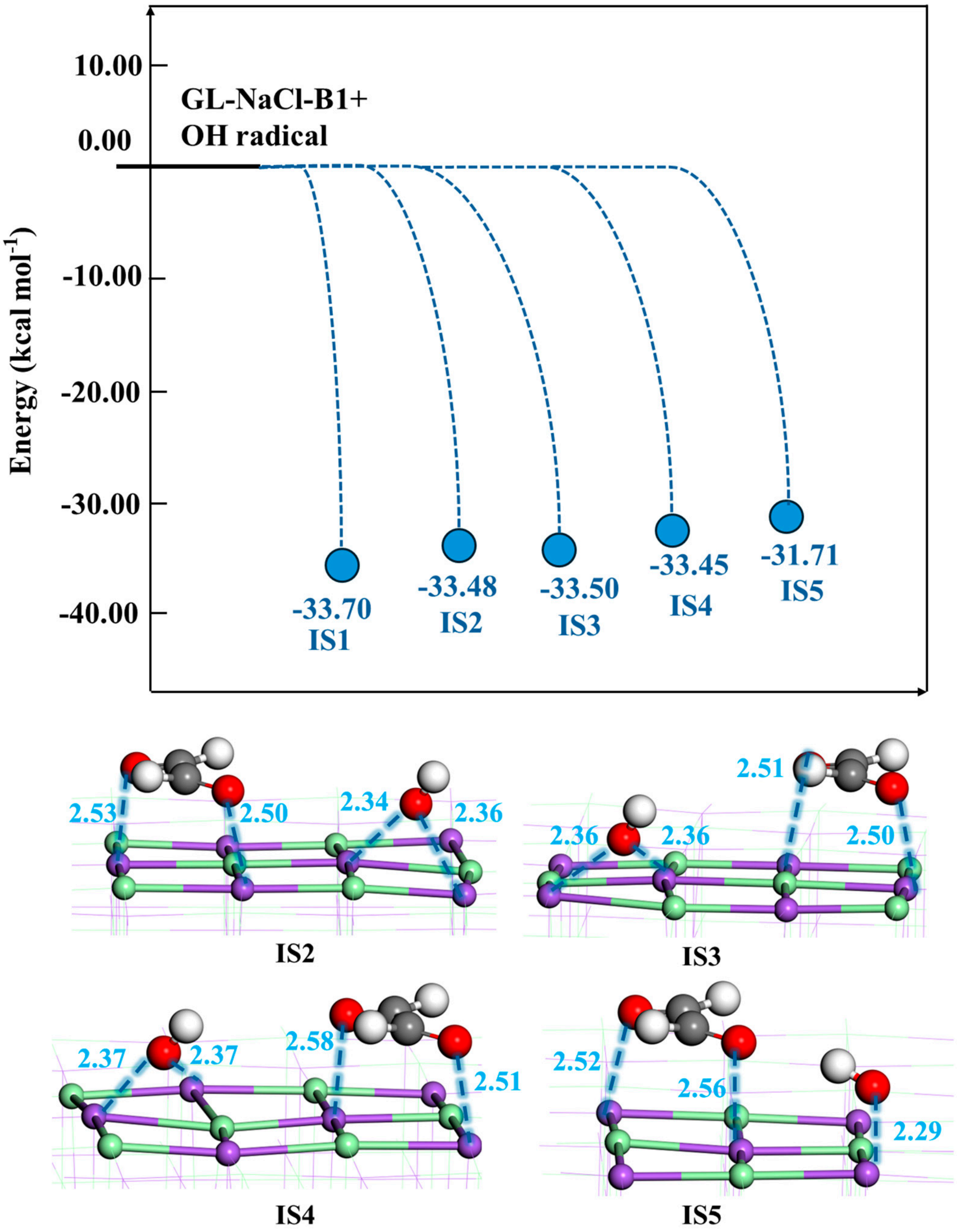

Figure S4. Potential energy surfaces for the formation of ISs along with the corresponding optimized geometries of ISs (bond lengths in Å).

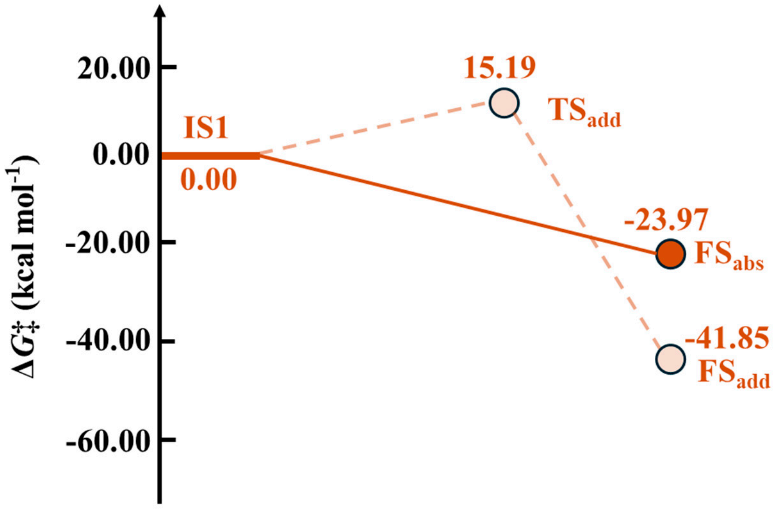

Figure S5. Gibbs free energy profiles for reaction pathways of GL and OH radical.
